# Gastrin: From Physiology to Gastrointestinal Malignancies

**DOI:** 10.1093/function/zqab062

**Published:** 2021-11-26

**Authors:** Suzann Duan, Karen Rico, Juanita L Merchant

**Affiliations:** Department of Medicine, Division of Gastroenterology and Hepatology, Arizona Comprehensive Cancer Center, University of Arizona, Tucson, AZ 85724, USA; Department of Medicine, Division of Gastroenterology and Hepatology, Arizona Comprehensive Cancer Center, University of Arizona, Tucson, AZ 85724, USA; Department of Medicine, Division of Gastroenterology and Hepatology, Arizona Comprehensive Cancer Center, University of Arizona, Tucson, AZ 85724, USA

**Keywords:** somatostatin, G-cell, hypergastrinemia, gastrinoma, MEN1, neuroendocrine tumor, GEP-NET, *Helicobacter pylori*

## Abstract

Abetted by widespread usage of acid-suppressing proton pump inhibitors (PPIs), the mitogenic actions of the peptide hormone gastrin are being revisited as a recurring theme in various gastrointestinal (GI) malignancies. While pathological gastrin levels are intricately linked to hyperplasia of enterochromaffin-like cells leading to carcinoid development, the signaling effects exerted by gastrin on distinct cell types of the gastric mucosa are more nuanced. Indeed, mounting evidence suggests dichotomous roles for gastrin in both promoting and suppressing tumorigenesis. Here, we review the major upstream mediators of gastrin gene regulation, including inflammation secondary to *Helicobacter pylori* infection and the use of PPIs. We further explore the molecular biology of gastrin in GI malignancies, with particular emphasis on the regulation of gastrin in neuroendocrine neoplasms. Finally, we highlight tissue-specific transcriptional targets as an avenue for targetable therapeutics.

## Discovery and Controversy

The earliest concept of a hormonal axis in the regulation of digestive physiology was borne from a series of experiments conducted in 1902 by English physiologists William Bayliss and Ernest Starling. Prior to their introduction, the prevailing doctrine on gastrointestinal (GI) secretory function was firmly established by Ivan Pavlov's 1897 publication *The Work of the Digestive Glands*.^1^ In direct opposition to Pavlov's assertion of a local nerve-centric mechanism in regulating the digestive response, Bayliss and Starling presented clear evidence of a circulating hormonal messenger (ie, secretin) that stimulated pancreatic secretory activity.^2^ Shortly thereafter, a series of seminal studies led by John Edkins elucidated an analogous mechanism in the stomach and contributed to the pivotal discovery of the acid-stimulating hormone known as gastrin.

Edkin's studies centered on venous injection of gastric mucous membrane extracts into anesthetized cats and subsequent evaluation of fluctuations in gastric acid secretion. In these experiments, Edkins noted that cats injected with pyloric extracts produced markedly elevated levels of gastric acid and pepsin compared to those injected with extracts prepared from the fundic mucosa. In his 1905 manuscript entitled *On the Chemical Mechanism of Gastric Acid Secretion*, Edkins communicated his observations and posited that an excitatory paracrine factor secreted by antral mucosal cells, which he termed gastrin, was responsible for activating secretory cells of the stomach during digestion.^3^ However, Edkin's claims were largely dismissed in favor of accruing evidence that supported a histamine-centric humoral mechanism for gastric motility and secretion that emerged from its discovery in 1910.^4^ For the remainder of his career, Edkin's theory on gastrin remained the target of substantial scrutiny from the scientific establishment. Consequently, a pro-secretory role for gastrin only began to emerge after his death in 1940.^5^

In 1938, Simon Komarov, a research assistant working under Boris Babkin at McGill University, successfully isolated an active preparation of gastrin from the pyloric mucosa.^6^ In 1942, Komarov published his work showing that the histamine-free concentrate could indeed stimulate acid secretion, thereby vindicating Edkin's initial report released nearly four decades prior. On the heels of this discovery, Roderic Gregory and Hilda Tracy further developed Komarov's early isolation techniques and identified a pair of heptadecapeptides, subsequently defined as gastrin I and II. Processing hundreds of porcine antra per week, Gregory and Tracy generated industrial volumes of the peptide and enlisted chemist George Kenner to perform the sequencing.^7^ As a result of these efforts, gastrin became the first GI peptide to have its complete molecular structure elucidated, thus laying the groundwork for further investigation into gastrin analogues and therapeutic antagonists.

## Gastrin Synthesis and Physiological Signaling

Gastrin is released by antropyloric G-cells in response to vagal, luminal, and hormonal stimuli. Central efferent vagal fibers permeating the gastric myenteric plexus stimulate the release of gastrin-releasing peptide (GRP) and vasoactive peptide (VIP) from neurons that innervate antropyloric G-cells.^8^ Mechanical distention from food ingestion stimulates vagal nerves, whereas the presence of digested peptides and amino acids in the lumen directly stimulate GRP-containing neurons.^9^ Additionally, peripheral mechanisms mediating gastrin release depend on the suppression of inhibitory signals, including somatostatin.^10^ D-cells within the pyloric antrum release somatostatin upon vagal and luminal stimulation following fasting and gastric acidification (pH < 3.0). Somatostatin inhibits gastrin in a paracrine fashion by binding to the transmembrane somatostatin 2 receptor (SSTR2), a G-protein coupled receptor expressed on neighboring antral G-cells.^11^ Conversely, meal ingestion inhibits somatostatin secretion via chemosensory signaling pathways mediated by acetylcholine release. While D-cells open to the lumen exist predominantly in the antrum, a smaller subpopulation of “closed” oxyntic D-cells exists in the corpus. Unlike their antral counterparts, these “closed” oxyntic D-cells lack luminal access, and thus respond exclusively to vagal stimulation and locally produced hormones, including GRP, CCK, and secretin.^12^

Gastrin primarily mediates its effects by binding to the cholecystokinin B (CCKB) receptor expressed on parietal cells and enterochromaffin-like (ECL) cells of the stomach. Activation of the G-protein coupled receptor generally stimulates phospholipase C and downstream calcium mobilization through protein kinase C activity.^13^ CCKB receptor-mediated activation of parietal cells directly stimulates the release of H^+^ ions through upregulation of H^+^/K^+^–ATPase.^14^ In contrast, gastrin-mediated activation of oxyntic ECL cells indirectly potentiates gastric acid secretion by releasing histamine, which in turn stimulates parietal cell acid secretion.^15^

## Gastrin As a Growth Factor

Gastrin has long been characterized as a trophic factor in both normal GI epithelial development and during neoplastic transformation. Indeed, gastrin is known to activate multiple mitogenic signal transduction pathways, including those mediated by the epidermal growth factor receptor (EGFR), phosphoinositide 3-kinase (PI3K), and MAPK activity.^16–19^ Prolonged hypergastrinemia resulting from dysregulated negative feedback mechanisms is associated with hyperplasia of the oxyntic mucosa. Disruption of acid-mediated gastrin inhibition leads to atrophic gastritis, sustained induction of gastrin gene expression, and expansion of ECL and parietal cell populations.^20,21^ Zollinger–Ellison syndrome occurs secondary to tumor-mediated hypergastrinemia in the absence of parietal cell atrophy. The resulting Type II carcinoids develop in response to gastrin stimulating proliferation of the ECL cells. Hypergastrinemia may also result from autoimmune gastritis, a chronic inflammatory syndrome in which autologous antibodies target and destroy the parietal cell (atrophic gastritis).^22^ These events preface the appearance of chronic achlorhydria and increased production of gastrin by antropyloric G-cells. Foveolar epithelial cell proliferation within the gastric pit coincides with a marked loss of parietal cells and reduced acid secretion (gastric atrophy), further potentiating gastrin gene expression. Subsequent gastrin-induced hyperplasia of ECL cells due to gastric atrophy supports the emergence of Type 1 gastric carcinoids that constitute a majority of gastrin-dependent tumors.^22^

## Gastrin in Gastric Stem Cell Differentiation

Hyperplastic lesions of the oxyntic mucosa exhibit low Ki-67 immunoreactivity, suggesting that gastrin-mediated mitogenic signaling favors underlying changes to stem cell differentiation in otherwise terminally differentiated parietal and ECL cell populations.^23,24^ In support of this, Wang and colleagues reported a role for gastrin in activating a population of CCKBR^+^ progenitor cells located in the proliferative isthmus of the gastric glands.^25,26^ Intriguingly, activation of CCKBR by amidated gastrin stimulates expansion of the stem cell pool in the gastric cardia and proximal corpus, while amidated gastrin exerts an inhibitory effect on CCKBR^+^ stem cells of the antrum.^26^ The distinct actions of gastrin on progenitor cells of the corpus and antrum correlate with the development of proximal gastric tumors and oxyntic hyperplasia.^27,28^ By contrast, gastrin-deficient mice exhibit a greater propensity for antral carcinogenesis.^29,30^ These dichotomous effects may, in part, be explained by the selective responsiveness of antral CCKBR^+^ stem cells to progastrin and insensitivity to pro-proliferative signaling effects mediated by amidated gastrin-17.^26^

**Figure 1.**
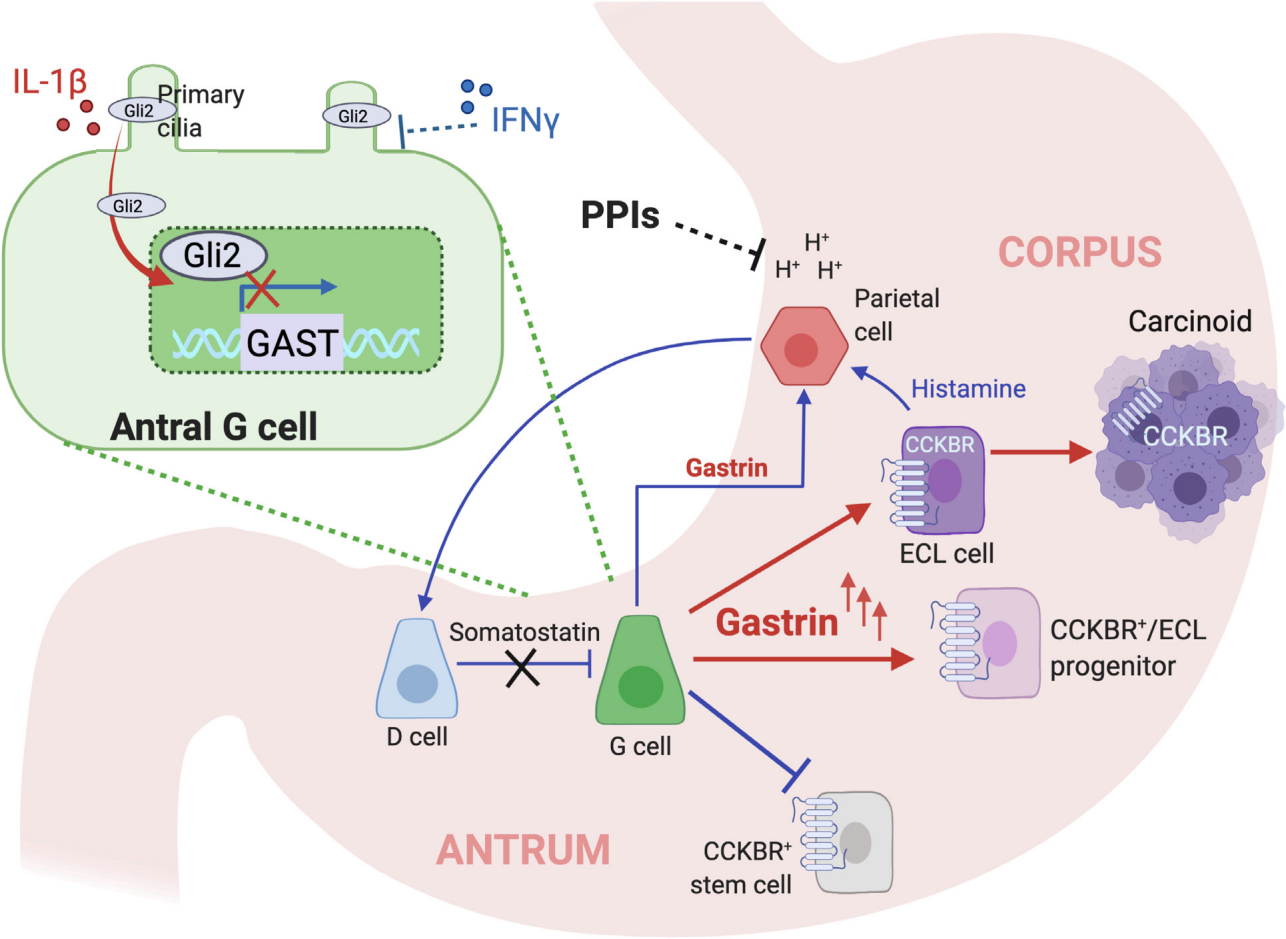
Mechanisms of gastrin signaling under physiological and specific pathological conditions. Under normal physiological conditions, gastrin participates in negative feedback regulation that involves acid-induced release of somatostatin from the antral D cell. Chronic inhibition of parietal cell acid secretion by proton pump inhibitors (PPIs), stimulates hypergastrinemia in human and mice. In genetically engineered mice exhibiting conditional loss of menin and somatostatin, PPIs can promote gastric carcinoid development. Gastrin stimulates enterochromaffin-like (ECL) cell proliferation through the cholecystokinin B receptor (CCKBR) and expands CCKBR + stem/progenitor cells *in the corpus*. By contrast in the antrum, gastrin inhibits the expansion of CCKBR + stem/progenitor cells. Therefore, gastrin's effects are likely site and context-dependent, eg, during chronic infection with *Helicobacter pylori*. *Helicobacterpylori*-elicited cytokines can positively or negatively regulate gastrin gene expression and antral hyperplasia by modulating GLI2 activation through primary cilia. Figure created with Biorender.com.

There remains a general consensus that ECL cell hyperplasia in the corpus arises from proliferation of the existing resident enteroendocrine cell (EEC) population as a direct result of elevated gastrin signaling. However, more recent evidence points to an alternative cellular target upstream of an expanding ECL cell pool. In these studies, mice receiving gastrin infusion or the proton pump inhibitor (PPI) omeprazole exhibited increased Ki-67 labeling of CCKBR^+^ progenitor cells near the gastric isthmus. These cells lacked any apparent expression of the ECL cell marker histidine decarboxylase. Subsequent lineage-tracing studies confirmed that CCKBR marks a short-lived population of immature ECL and parietal cells, which expand in response to hypergastrinemia and serve as a reservoir for mature ECL cells with reduced proliferative potential (Figure 1).^31^

The Sonic hedgehog (Shh) signaling pathway has more recently emerged as a player in gastric cancer progression, with mounting evidence of aberrant Shh signaling during *Helicobacter pylori*-mediated inflammation and tumorigenesis. Shh is expressed in all major cell lineages of the corpus,^32^ and is required for maintaining the gastric mucosa by controlling epithelial cell proliferation and apoptosis. Gastric atrophy accompanied by *H. pylori* infection coincides with the loss of Shh expression in parietal cells.^32,33^ Further, parietal cell-specific deletion of Shh stimulates hypergastrinemia and hyperplasia of surface mucous cells in transgenic mice.^34^ In the healthy adult stomach, gastrin regulates Pepsin A-mediated proteolytic processing of Shh peptide into its active form through its ability to induce gastric acid.^33^ However, Shh processing was inhibited in the hypochlorhydric stomach due to parietal cell atrophy that precedes gastric cancer.^33^ Furthermore, a direct role for gastrin in regulating gastric epithelial architecture is supported by evidence of gastrin-mediated induction of Indian hedgehog (Ihh) expression and surface epithelial proliferation in the gastric mucosa of mice lacking parietal cell-specific Shh expression.^35^ Collectively, these studies expand on a potential Hedgehog-dependent mechanism for gastrin-mediated proliferation of the gastric epithelium, creating an environment supportive of neoplastic development.

Chronic infection with *H. pylori* and widespread usage of proton pump inhibitors (PPIs) have been extensively studied as a cause of hypergastrinemia secondary to atrophic gastritis. *Helicobacter pylori* infection is associated with a 9-fold increase in gastric cancer risk, particularly in the distal stomach.^36^ In recent years, Western nations have seen a dramatic shift in the location of gastric adenocarcinoma from the distal antrum to the proximal stomach.^37,38^ Tumors arising in the proximal stomach tend to be poorly differentiated, implicating deregulation of mitogenic signaling and stem cell differentiation pathways that support normal gastric cell specification.^39^ PPI-induced hypergastrinemia use has been speculated to play a potential role in this epidemiological shift.^40,41^ Indeed, studies in mice suggest a growth-promoting role for gastrin that synergizes with other cofactors or mutant phenotypes.^42^ Furthermore, recent independent and large-scale population studies suggest a link between PPI use and elevated gastric cancer risk.^43–46^

## Mechanisms of Gastrin Signaling in Adenocarcinoma

The gastric epithelium undergoes constant renewal that requires the integration of intrinsic and non-cell autonomous regulatory cues to maintain homeostasis. Thus, perturbations of normal growth patterns and programmed cell death machinery may contribute to neoplastic transformation. In addition to its role in stimulating gastric stem cell activation and epithelial proliferation, gastrin exerts both antiapoptotic and mitogenic signaling in various GI malignancies. These effects are largely mediated through activation of CCKBR, known to be upregulated in human gastric neuroendocrine neoplasms^47^ and gastric,^48–51^ pancreatic,^52–54^ and colorectal adenocarcinomas.^55,56^ Gastrin exerts a direct trophic effect on gastric cancer cells in vitro and stimulates the growth of human colorectal and gastric cancer xenografts through a CCKBR-dependent mechanism.^57–59^ Moreover, gastrin stimulates downstream pro-proliferative pathways, including those mediated by β-catenin/cyclin D1^60,61^ and the EGF receptor.^62^ In the latter mechanism, gastrin-mediated activation of CCKBR transactivates EGFR via PKC signaling, and these events converge on the heparin-binding (HB)-EGF promoter through a gastrin-responsive *cis*-acting regulatory element to stimulate cell proliferation.^63^

An antiapoptotic role for gastrin has been demonstrated across multiple studies employing in vitro and in vivo models of tumorigenesis. Genome-wide microarray analysis of a rat pancreatic adenocarcinoma cell line revealed significant induction of pro-survival genes following sustained treatment with gastrin, and these events coincide with a PKC-dependent reduction in caspase-mediated apoptosis.^64^ Consistent with this report, elevated expression of the pro-survival protein clusterin has been observed in rodent models of hypergastrinemia as well as in human biopsies of gastric adenocarcinoma and carcinoids.^65,66^ Moreover, gastrin-induced clusterin expression was reported to exert a cyto-protective effect by driving resistance to starvation and chemotherapy-induced cell death.^66^ Concomitantly, gastrin was reported to modulate the activity of the antiapoptotic BCL-2 signaling pathway and stimulate cell proliferation by upregulating the expression of MCL-1,^67^ BCL-2, and BAK.^68,69^ For example, gastric biopsies from 10 patients with gastric carcinoids and hypergastrinemia showed positive immunoreactivity for MCL-1 and this correlated with low expression of the apoptotic marker cleaved caspase-3 in regions of ECL cell hyperplasia.^67^

While these studies support a role for gastrin in modulating cytoprotective pathways leading to proliferation and resistance to chemical stress, the mechanisms that regulate gastrin gene expression in response to these conditions remain poorly defined. To address this, Westwood and colleagues demonstrated a context-specific role for HIF1α in regulating gastrin expression under conditions of hypoxia. Here, HIF1α binds the gastrin promoter to induce gastrin gene expression, leading to enhanced resistance to hypoxia-induced apoptosis.^70^ Interestingly, Wang and colleagues identified opposing effects of gastrin on various gastric cell types leading to cell proliferation or alternatively, apoptosis.^71,72^ Using the INS-GAS mouse model, the authors demonstrated a cytotoxic role for gastrin in stimulating apoptosis of parietal cells, extraglandular stromal cells, and infiltrating immune cells. These events were concomitant with high cellular turnover and an increased density of gastric pit cells preceding carcinogenesis.^72^

## Gastrin During *H. pylori* Infection

In 1989, Calam and colleagues introduced the “gastrin link hypothesis” and suggested that hypergastrinemia resulting from *H. pylori* infection directly supports ulcerations in the duodenum.^73^ This concept was further refined to elucidate two distinct pathophysiological outcomes resulting from *H. pylori* infection in the stomach. Generally, patients infected with *H. pylori* exhibit 2–3-fold higher fasting gastrin levels and elimination of the infection has been shown to restore basal gastrin expression.^73–75^ These events are primarily supported by a reduction in CCK and D-cell-mediated release of somatostatin, thus resulting in impaired normal gastrin inhibitory mechanisms.^76^

The response of the oxyntic mucosa to elevated gastrin levels operates as a defining feature in determining the pathophysiological outcome. Hypergastrinemia resulting from antral-dominant gastritis stimulates the proliferation of parietal cells and enhances acid secretion. This creates an ulcer-prone environment in which the pH neutralization processes in the duodenum are overwhelmed.^77^ In contrast, non-ulcer patients presenting with corpus-dominant or pangastritis exhibit reduced oxyntic sensitivity to gastrin (2-fold reduction), likely as a result of widespread inflammation in the gastric body.^78^ As a consequence, *H. pylori* infection results in achlorhydria and promotes atrophic gastritis, bacterial overgrowth, and gastric metaplasia, a microenvironment predisposing to gastric cancer.^79–81^

Hypergastrinemia underlying *H. pylori* infection has been explored extensively in vitro, beginning with reports of gastrin secretion by canine antral G-cells following direct exposure to *H. pylori*.^82,83^ Further work demonstrated that the *H. pylori*-elicited cytokines IL-8, IL-1β, and TNFα stimulate canine antral G-cells and human antral biopsy fragments to release gastrin.^84,85^ Colonization of the gastric antrum by *H. pylori* is known to induce a Th1/Th17 response that coincides with an increase in gastrin secretion and prefaces gastric atrophy and intestinal metaplasia. Mechanistically, IFNγ, a classical Th1 cytokine, and IL-1β are thought to play a role in this process as both cytokines are upregulated following gastric infection. Translating these observations in vivo has proved challenging, as mice exhibit a corpus-dominant phenotype following infection with *Helicobacter sp*., while infection in humans tends to be antral-predominant. To address this, an increasing number of mouse models have been generated with tissue-restricted expression of cytokines downstream of infection with *Helicobacter sp*. such as *Helicobacterfelis*. Using this approach, we recently showed that the downstream signaling effects of specific *Helicobacter*-elicited cytokines are more nuanced and likely reflect the temporal progression of inflammatory signaling during gastric infection. Whereas, directing IFNγ expression to the antrum in mice increased gastrin expression and stimulated antral hyperplasia, overexpressing IL-1β resulted in reduced gastrin levels but also coincided with antral hyperplasia. Mechanistically, IFNγ-mediated induction of gastrin was found to occur through suppression of Gli2, a repressor of gastrin gene expression and mediator of Shh signaling. In contrast, IL-1β induced Gli2 expression and suppressed gastrin expression through modulation of primary cilia length on gastrin-expressing cells. These observations support a critical role for primary cilia in transducing upstream IL-1β signaling in the regulation of gastrin expression, ultimately leading to loss of endocrine cells types in favor of epithelial hyperplasia. Importantly, these studies highlight opposing effects of *Helicobacter*-elicited cytokines in regulating gastrin expression (Figure 1).^86^ It should be noted that primary cilia mediate other GPCR such as SSTR3,^87–89^ which might have relevance for understanding somatostatin inhibition of the G-cell.

Additionally, direct mechanisms of *H. pylori*-mediated gastrin regulation have also been proposed, with conflicting evidence to support a role for the *H. pylori* virulence factor cytotoxin-associated protein A (CagA) in regulation of the gastrin promoter. In human gastric cancer cells, infection with *H. pylori*/CagA^+^ induces gastrin mRNA through a MEK/ERK and JAK-dependent mechanism.^90,91^ However, previous studies by our group show that the CagA element is dispensable for gastrin gene activation.^92^ Interestingly, CagA^+^*H. pylori* infection has also been reported to exert epigenetic regulation of the gastrin promoter through a genome-wide decrease in methylation at CpG sites.^93^ Expanding our understanding of *H. pylori*-induced hypergastrinemia has revealed a synergistic relationship that may contribute to the development of gastric metaplasia and predisposition to cancer.^94^

## Regulation of Gastrin Gene Expression

G-cell extrinsic regulatory cues that modulate gastrin gene expression include paracrine regulation by D-cells and stimulatory ligands that are produced locally or during bacterial infection. While gastric acidity is a known stimulus for gastrin release, fluctuations in pH indirectly regulate gastrin gene expression through activation of D-cell-mediated release of somatostatin. Other factors known to regulate secretion but not expression of gastrin include the peptides GRP and bombesin. Early studies intended for screening gastrin regulatory factors identified epidermal growth factor (EGF) receptor ligands as direct modulators of gastrin gene expression in both human and rat endocrine tumor cell lines.^95,96^ Subsequently, a 16 bp GC-rich EGF response element (gERE) was mapped to the human gastrin promoter and Sp1 was subsequently shown to bind this element.^97^ A physiological role for the gERE is further supported by the presence of EGF receptor ligands in the stomach, produced locally either through a parietal cell-mediated response to hypergastrinemia^98,99^ or via the immune compartment during acute and chronic inflammation.^100–102^

Several DNA regulatory elements have been mapped to the gastrin promoter and include both tissue-specific and inducible elements. Tissue-specific regulatory elements, specifically a homeodomain, CACC, and gastrin negative element were mapped to 450 bp of the human gastrin promoter and the first exon.^103^ In contrast, inducible and basal regulation of gastrin gene expression by EGF, cAMP, and inflammatory cytokines are thought to require only the first 240 bp of the gastrin promoter.^95,104^ In addition to the gERE, Sp1 was observed to bind the CACC element, as well as to another GC-rich element upstream of the gERE to regulate gastrin gene expression.^105^ The transactivating function of Sp1 is opposed by the recruitment of ZBP-89, a Kruppel-type four zinc finger transcription factor that also binds to the gERE and acts to repress gastrin expression.^106^ Additional signaling factors and pathways have been reported to synergize and converge on Sp1 binding to the gastrin promoter. For instance, constitutive activation of the Ras-Erk pathway, such as that observed in *K-ras*-mutated colon cancers, induces phosphorylation of Sp1 and enhances its binding affinity to the human gastrin promoter.^107^ Interaction of Sp1 with AP-1 transcription factor family members at the proximal gastrin promoter has also been reported. In chromatin immunoprecipitation studies, Sp1 and JunD were shown to cooperate at the Sp1 and gERE binding sites and drive gastrin transactivation. Notably, JunD was also observed to bind a non-consensus AP-1 site within the proximal promoter, suggesting direct regulation of gastrin by JunD independent of Sp1 binding.^107^

These findings provide a link between the emergence of MEN1 gastrinomas and the role of the tumor suppressor protein menin in regulating gastrin gene expression. Loss of menin, either in the context of the MEN1 syndrome or resulting from sporadic mutations within the *MEN1* locus, is associated with the development of gastroenteropancreatic neuroendocrine tumors (GEP-NETs). Previous work by our group has identified a role for menin in repressing gastrin gene expression by disrupting the association of JunD and Sp1 with their respective regulatory promoter elements.^108,109^

Other transcriptional regulators of gastrin include the zinc finger transcription factor GLI2. Hedgehog (Hh) signaling renders GLI2 transcriptionally active in the nucleus, where it has been shown to bind the gastrin promoter and regulate downstream gene expression.^110^ Constitutive activation of GLI2 in the gastric epithelium was shown to suppress gastrin expression and promote antral cell proliferation leading to hyperplasia.^110^ These observations suggest a critical role for the hedgehog signaling pathway in mediating feedback regulation of gastric acid secretion and may potentially explain the discordant effects of gastrin in the corpus and antrum.

## Gastrinomas

In contrast to overall declining cancer incidence rates, GEP-NETs have seen a 6-fold upsurge in incidence since the 1970s.^111^ Similarly,  the prevalence of GEP-NETs continues to climb, placing these malignancies among the most prevalent digestive cancers in the United States.^112^ GEP-NETs are physiologically complex neoplasms and include gastric carcinoids, gastrinomas, and pancreatic neuroendocrine tumors. In recent years, a substantial effort to characterize the signaling mechanisms that underlie these malignancies has shed light on their unique origins, mutational signatures, and clinical features.

## The Tumor Suppressor Protein Menin in Gastrinoma Pathogenesis

GEP-NETs are commonly associated with sporadic and inherited mutations in the Multiple Endocrine Neoplasia type I (*MEN1*) gene. Consistent with Knudson's “two-hit” hypothesis,^113^ the autosomal dominant condition is characterized by an acquired germline mutation in one *MEN1* allele, followed by loss of the second allele within the tumor by deletion (loss of heterozygosity) or inactivating point mutations.^114^ Patients presenting with the MEN1 syndrome experience a higher risk for developing multiple endocrine tumors in the pancreas, pituitary, and upper GI tract. In addition, patients carrying a *MEN1* mutation are predisposed to developing GI NETs that produce excess levels of gastrin.^115^ Such MEN1-associated gastrinomas preferentially develop in the submucosa of the duodenum, are small (<1 cm), multiple, and metastatic.^116^

Inactivation of *MEN1* as a result of frameshift, missense, and nonsense mutations causes loss of the tumor suppressor protein menin. Menin is a highly conserved and ubiquitously expressed nuclear scaffold protein that complexes with multiple transcription factors to regulate downstream target gene expression. Known transcriptional binding partners include the Mixed Lineage Leukemia proteins (MLL1 and MLL2),^117,118^ NF-κB,^119^ and the AP-1 transcription factor JunD among others.^120,121^ In endocrine cells, menin represses transcriptional activation of various gene targets involved in supporting cell growth and proliferation, including gastrin. For instance, menin-mediated interaction with JunD represses its function as a transactivator of gastrin gene expression.^107^ Therefore, loss of nuclear menin function in gastrin-expressing G cells is thought to be an essential event underlying the formation of MEN1 gastrinomas.

Despite the identification of over 1200 germline modifications, *MEN1* mutations do not correlate with specific phenotypes.^122,123^*MEN1* mutations appear to be scattered throughout the gene locus and lack any apparent mutational hotspots.^122^ Moreover, individuals within the same family that carry identical mutations may exhibit disparate phenotypes.^124^ The most common *MEN1* mutations are frameshift deletions or insertions (41%), followed by nonsense mutations (23%), missense mutations (20%), splice-site mutations (9%), in-frame deletions or insertions (6%), and whole or partial gene deletions (1%).^122^ Nevertheless, most of these mutation studies were performed in non-GEP-NETs. Therefore, the diversity among *MEN1* mutations in tissue location and phenotype has precluded the full characterization of *MEN1* mutations in GEP-NETs. Intriguingly, the majority of *MEN1* gastrinomas originate from hyperplastic G cells that retain a functional *MEN1* allele.^125–127^ This observation suggests the possibility of alternative mechanisms resulting in loss of menin function independent of *MEN1* gene inactivation. For example, studies of *MEN1* gastrinomas have identified mutations in the *MEN1* locus leading to aberrant nuclear translocation of menin as well as accelerated protein degradation.^128–130^

*In vivo* models of GEP-NET pathogenesis are historically lacking, in part due to tissue heterogeneity from which neoplasms arise, and the absence of known driver mutations preceding malignancy.^131^ Nevertheless, the 21st century has seen an expansion in the number of transgenic mouse models aimed at clarifying the role of *MEN1* and other putative drivers in GEP-NET emergence.^132–134^ Francis Collin's group at the National Human Genome Research Institute was among the first to recapitulate the human MEN1 syndrome through homologous recombination of the *Men1* locus. Homozygous deletion of *Men1* in murine embryonic stem cells results in embryonic lethality, whereas heterozygous inactivation coincides with multiple clinical features of the human MEN1 syndrome. Notably, while heterozygous mice develop endocrine tumors of the pancreatic islets and pituitary similar to those observed in patients, no gastrinomas were reported in this model.^135^ Subsequent mouse models generated by our group addressed the absence of any apparent gastric phenotype by conditionally deleting *Men1* from the GI tract epithelium. Expressing *Cre recombinase* from the *Villin* promoter deleted the *Men1* locus in intestinal epithelial cells (*Men1^Δ^^IEC^*) and resulted in antral G-cell hyperplasia and hypergastrinemia.^108^ Removal of the somatostatin-mediated feedback regulation by breeding the *Men1^Δ^^IEC^* mice onto a somatostatin null background (*Men1^Δ^^IEC^: Sst^–^^/^^–^*) led to significant hypergastrinemia and the development of gastric carcinoids.^136^ These events were accelerated following systemic gastric acid suppression using the PPI omeprazole. A total of 6 mo of omeprazole treatment was sufficient to synergistically stimulate the development of G-cell hyperplasia in the proximal duodenum of *Men1^Δ^^IEC^: Sst^–^^/^^–^* mice.^109^ Collectively, these studies confirmed the ability of menin to suppress gastrin.

## Molecular Heterogeneity of GEP-NETs

Understanding the mutational profile of GEP-NETs is essential to uncovering key driver mutations that can be therapeutically targeted. Previously, 48 small intestinal neuroendocrine neoplasms consisting mainly of carcinoids were analyzed by whole exome sequencing (WES).^137^ While a mutation in the cell-cycle inhibitor *CDKN1B* was found in a small population of tumors (8%), no common somatic mutations were shared amongst other GEP-NETs. Consistent with other reports, small intestinal neuroendocrine neoplasms (SI-NENs) such as ileal carcinoids, which arise from serotonin-secreting enterochromaffin cells, present with limited somatic driver mutations and are considered amongst the most genetically stable cancers.^131,137,138^ Thus, more promising avenues toward precision medicine may lie in targeting genomic instability and aberrant methylation phenotypes. Indeed, both hypermethylation of select genomic loci^139^ and increased frequency of chromosomal losses (eg at the terminal end of chromosome 18q) have been observed in SI-NENs.^140,141^

In contrast to SI-NENs, large-scale molecular profiling identified recurrent spontaneous mutations in pancreatic neuroendocrine tumors (PNETs). For instance, WES analysis of 98 PNETs showed recurrent somatic mutations in *MEN1* (44%), alpha thalassemia/mental retardation syndrome X-linked (*ATRX*; 18%) and death domain-associated protein (*DAXX*; 25%).^142^ Inactivating mutations in ATRX and DAXX are associated with altered telomeres,^143^ chromosomal instability, and reduced survival in patients with PNETs.^144^ In addition, the presence of chromosomal instability is well-established in PNETs.^145,146^ For example, 40% of patients with PNETs have deletions in the 16p chromosome region,^147^ and loss of TSC2 at this site is implicated in deregulation of the PI3K/AKT/mTOR pathway.^138^ Furthermore, the methylation profile of PNETs differs from that of SI-NENs, suggesting fundamental differences in pathogenesis.^139^ Indeed conditional deletion of *Men1* and *Pten*, the inhibitor of the PI3K/AKT/mTOR pathway, induces both pancreatic and pituitary neuroendocrine tumors and confirms cooperation between the two loci.^148^

**Figure 2.**
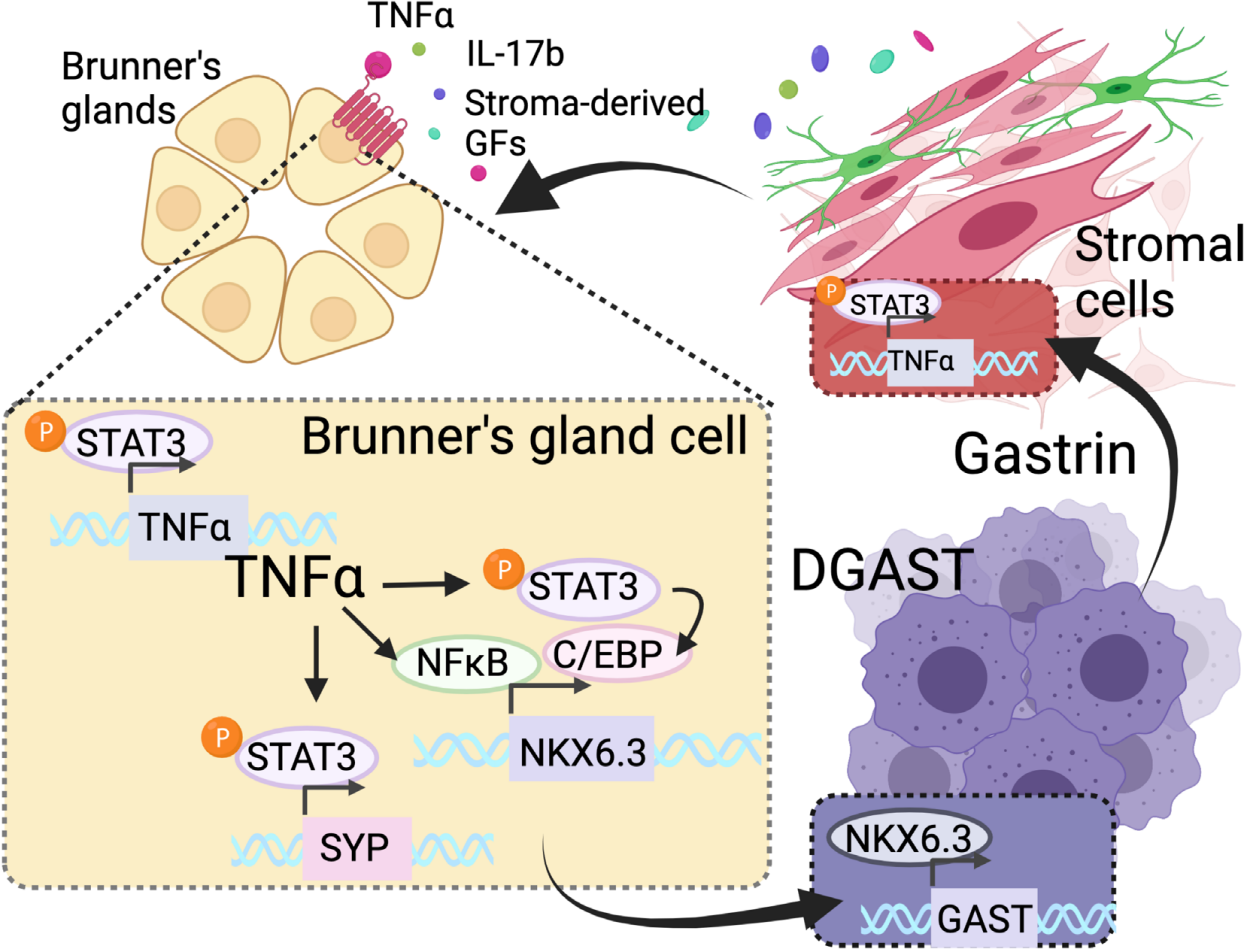
Proposed model of cytokine-elicited epithelial reprogramming events that precede gastrinoma development in the duodenum. Duodenal gastrinomas (DGAST) arise within the Brunner's glands of the proximal duodenum,^116^ raising the likelihood that this hormone producing tumor arises from a reprogrammed cell and does not arise directly from enteroendocrine cells. Here, we propose that stromal-derived inflammatory cytokines, such as TNFα or IL-17, activate STAT3 phosphorylation and NFκB signaling pathways that reprogram the Brunner's glands toward a neuroendocrine phenotype. STAT3 and NFκB signaling induce transcription factor NKX6.3, a homeobox transcription factor required for gastrin gene expression and master regulator of gastric differentiation.^153,154^ Figure created with Biorender.com and adapted from Rico *et al*. (2021), *BMJ Open Gastroenterology*.^149^

To identify transcriptional targets unique to duodenal gastrinomas (DGASTs), we recently reported on a genome-wide analysis of surgically resected DGASTs and PNETs. In these studies, RNA-sequencing revealed an enrichment of IL-17 and TNF⍺ signaling pathways in DGASTs, however digital spatial profiling of tumors and the adjacent Brunner's glands confirmed a scarcity of immune cells within the tumor. Immunofluorescent analysis indicated strong immunoreactivity of tumor cells, Brunner's glands, and the tumor stroma for both cytokines and downstream pSTAT3 activation.^149^ Both IL-17 and TNF⍺ are known to activate downstream targets through NF-κB and pSTAT3 signaling pathways. Furthermore, previous studies have shown that STAT3 binds the SYP promoter, suggesting a direct mechanism for cytokine-induced neuroendocrine reprogramming.^150–152^ In support of this, treatment of normal human duodenal organoids with TNF⍺ stimulated NF-κB and pSTAT3 activation and these events coincided with increased expression of neuroendocrine transcripts *SYP, CHGA*, and the gastrin-specification factor *NKX6.3*.^149,153,154^ Cytokine-mediated regulation of *NKX6.3* is underscored by *in silico* analysis identifying an NF-κB binding site in the 5’ UTR of the *NKX6.3* promoter. Taken together, these observations suggest a role for inflammatory cytokines in potential reprogramming of the Brunner's glands in favor of neuroendocrine differentiation and tumorigenesis (Figure 2).

## Pancreatic and DGASTs: Differing Cellular Origins?

Accruing evidence suggests diverging mechanisms of pathogenesis in gastrinomas arising from the duodenum and pancreas. It was previously reported that patients with Zollinger–Ellison syndrome and MEN1-related DGASTs had proliferative and hyperplastic gastrin cells in the nontumorous duodenum (i.e mucosal crypts and Brunner's glands). In contrast, no proliferative gastrin cell lesions were identified in patients with sporadic non-MEN1-based gastrinomas.^116^ Unlike the duodenal neuroendocrine tumors, hyperplastic G-cells did not exhibit LOH of the *MEN1* locus, thus implicating them as potential precursor lesions to DGASTs.^116^ However, the genetic and environmental stimuli that induce the transition of hyperplastic gastrin cells into tumors remains to be elucidated.

Generation of Men1*^Δ^^IEC^: Sst^–^^/^^–^* mice led to the first report of a genetically engineered mouse model to display gastric carcinoids.^136^ Introduction of PPI-mediated gastric acid suppression in these mice resulted in the emergence of hyperplastic gastrin-expressing cells within the lamina propria of the duodenum. Intriguingly, these gastrin-positive cells were not of epithelial, neuronal, or smooth muscle origin. Rather, the gastrin positive cells were found to express markers of mucosal enteric glial cells (EGCs). Moreover, gastrin expression by EGCs required a loss of menin.^109^ In the enteric nervous system, EGCs constitute a significant cell population found in the enteric ganglia between the smooth muscle layers and within the lamina propria. EGCs express the same markers as astrocytes in the CNS, such as Glial Fibrillary Acidic Protein (GFAP), p75^NTR^, and S100B protein. Additionally, EGCs express Sry-related HMG-Box gene 8 (SOX8), SOX9, and SOX10, all of which are expressed in multipotent progenitor cells of the enteric nervous system. The selective expression of various neuronal markers further defines EGC subpopulations.^155^ Recent application of a single-cell sequencing approach identified an EGC transcriptome signature consisting of *Sox10*, Erb-B2 receptor tyrosine kinase 3 (*Erbb3*), Fatty acid binding protein 8 (*Fabpp*), and Proteolipid protein 1 (*Plp1*).^156^

As EGCs of *Men1^Δ^^IEC^: Sst^–^^/^^–^* mice express gastrin, Sundaresan *et al*. used immunohistochemistry staining to examine whether human DGASTs also exhibit these markers.^96^ Surprisingly, human DGASTs (4/5) and lymph node gastrinomas (2/2) stained for EGCs markers while pancreatic gastrinomas (5/6) did not, raising the possibility of diverging cellular origins for duodenal and pancreatic gastrinomas.^109^ Indeed, DGASTs present with unique clinicopathologic features, eg, they are multiple, small (< 1 cm), and are more likely to metastasize to the lymph nodes.^157^ Since DGASTs express EGC markers, it remains plausible that hyperplastic G-cell lesions may differentiate from neural crest cells rather than from endoderm-derived epithelial cells, eg, EECs, as previously suggested.

EECs comprise approximately 1% of intestinal mucosal cells and function as mediators of paracrine and distant cell-to-cell communication. EECs express a variety of neuronal protein markers, in addition to neurotrophin receptors including the glial-derived neurotrophic factor (GDNF) receptor.^158^ Neuroendocrine cells are broadly identified by the secretion of Chromogranin A (CgA) or Chromogranin B (CgB), two key proteins that modulate neuroendocrine secretory function. Synaptophysin, a component of the presynaptic vesicle membrane, and the neural cell adhesion molecule CD56 (NCAM) are also signature proteins expressed by EECs. Furthermore, EECs represent a unique class of cells as they respond to both hormonal and neuronal signals.^158^

As EECs exhibit both neuronal and endocrine markers, there remains some controversy as to whether neuroendocrine cells develop from the endoderm or neural crest. Lineage tracing experiments using the *Lgr5+*-CreERT2 transgene and the Rosa26R-LacZ reporter demonstrate that all epithelial cells, including neuroendocrine cells of the intestinal mucosa, originate from *Lgr5 + *pluripotent stem cells.^159^ Previous embryologic studies using chick-quail chimeras confirmed that ganglion cells of the submucosa and myenteric plexus of the GI tract express neural crest markers, while mucosal EECs did not.^160^ The absence of neural crest markers in EECs suggested that GI neuroendocrine cells originated from the endoderm and, therefore, the epithelium. While substantial evidence suggests that neuroendocrine cells in the GI tract develop from the endoderm, it remains unknown whether neural crest cells can undergo context-specific modification, eg, acquired mutations in *MEN1*, and give rise to neuroendocrine cells with hormone-secreting capabilities. Hopefully, applying newer approaches of molecular profiling to GEP-NETs will illuminate our understanding of these heterogenous tumors, which invariably depend on cell location and cell type.

## Summary

The mitogenic actions of gastrin on ECL cells have long been established, however, more recent evidence suggests additional roles for gastrin signaling in activation of other cell types. Among these, gastrin has been reported to activate progenitor cells residing in the gastric isthmus of the proximal and distal stomach, leading to increased proliferation or asymmetric cell division. Further investigation into the cellular targets of gastrin signaling is needed to inform the potential effects of hypergastrinemia secondary to PPI use and infection by *H. pylori*. Pathological levels of circulating gastrin are perhaps best studied in the context of gastrin-producing GEP-NETs. GEP-NETs represent diverse neoplasms that vary in location, mutational profile, and response to therapy. The nonstochastic occurrence and invasive characteristics of these neoplasms suggest reprogramming of resident differentiated cell populations by the unique tissue microenvironment where GEP-NETs originate. For instance, up to 60% of DGASTs develop within mucous-producing Brunner's glands located in the proximal duodenum.^161^ Importantly, Brunner's glands provide a rich source of pro-proliferative growth factors, including EGFR ligands, and thus, may potentiate neoplastic transformation and pro-tumorigenic signaling within the duodenal microenvironment.^161–165^ Taken together, this knowledge suggests fundamental differences in the cellular origin and etiology of DGASTs compared to NETs arising from other tissues. In support of this, recent evidence presented by our group challenges the long-standing belief that hyperplastic gastrin-producing cells within the proximal duodenum originate from epithelial-derived EECs. In these transgenic mouse studies, neural crest-derived EGCs were implicated as potential cellular precursors to MEN1-related gastrinomas, thus shifting the current paradigm on the cellular origin of these cancers.

## Funding

This work has been supported by the Public Health Service Grant: R01 DK45729-27 to JLM.
